# Plasma Fatty Acids Pattern and Dry Eye Disease in the Elderly: The Montrachet Population-Based Study

**DOI:** 10.3390/nu14112290

**Published:** 2022-05-30

**Authors:** Alassane Seydou, Louis Arnould, Pierre-Henry Gabrielle, Florian Baudin, Ines Ben Ghezala, Alain M. Bron, Niyazi Acar, Catherine Creuzot-Garcher

**Affiliations:** 1 Ophthalmology Department, Dijon University Hospital, 21000 Dijon, France; alassanemaiga7@yahoo.fr (A.S.); phgabrielle@gmail.com (P.-H.G.); florian.baudin@chu-dijon.fr (F.B.); ines.ben-ghezala@chu-dijon.fr (I.B.G.); alain.bron@chu-dijon.fr (A.M.B.); catherine.creuzot-garcher@chu-dijon.fr (C.C.-G.); 2 Centre des Sciences du Goût et de l’Alimentation, AgroSup Dijon, CNRS, INRAE, Université Bourgogne Franche-Comté, 21000 Dijon, France; niyazi.acar@inrae.fr; 3 INSERM, CIC1432, Clinical Epidemiology Unit, Dijon University Hospital, 21000 Dijon, France

**Keywords:** dry eye disease, elderly, epidemiology, fatty acids pattern, lipids, population-based study, ocular surface

## Abstract

(1) Background: To investigate the association between plasma fatty acids (FAs) and dry eye disease (DED) in an elderly population; (2) Methods: We conducted a population-based study, the Montrachet study, in individuals older than 75 years. DED was evaluated using the Schirmer I test without anesthesia, tear film breakup time (TFBUT) measurement and fluorescein corneal staining. Plasma FAs were measured in fasting blood using gas chromatography; (3) Results: A total of 740 subjects with a plasma measurement of 25 FAs were included in this study. The mean age was 82.2 ± 3.7 years, and 62.7% were women. DED was present in 35.0% of participants. We identified a plasma FAs pattern positively associated with DED, characterized by low polyunsaturated fatty acids (PUFAs), high monounsaturated fatty acids (MUFAs) and low saturated fatty acids (SFAs) levels. After adjustment for major confounders, individuals in the upper quartile of the FAs pattern scores compared with those in the lower quartile were more likely to present DED (OR 2.46 (95% CI 1.51–4.01), *p* = 0.001); (4) Conclusion: In this study, we found that a plasma FAs pattern characterized by low PUFAs, high MUFAs and low SFAs was significantly associated with DED in elderly participants.

## 1. Introduction

Dry eye disease (DED) is one of the most common ocular surface diseases in the elderly [1]. DED prevalence rates range from 4.3% to 34.5%, depending on the age group and definition [2,3,4,5,6]. DED leads to discomfort and visual disturbances, impacting the quality of life of patients [7]. DED presents with two types of clinical manifestations: tear deficiency dry eye and evaporative dry eye, which leads to tear impairment and tear lipid layer instability [8]. These disturbances are more frequent in women than in men [9]. Several risk factors were shown to be associated with DED, including age, gender, smoking, autoimmune diseases, corneal dysfunction, menopausal status and environmental factors such as light and sun exposure and some medications [3,8].

Oxidative stress has been hypothesized to play a key role in DED by producing reactive oxygen species that induce damage on corneal and conjunctival epithelial cells and promote ocular surface inflammation [10,11]. Hyperosmolarity and inflammation also play a key role in the pathogenesis of DED, making DED to be considered as a chronic inflammatory disease [12]. Among inflammatory mediators, polyunsaturated fatty acids (PUFAs) have been associated with different ocular diseases involving inflammation pathways [13,14]. PUFAs have been used through dietary, systemic or topical delivery as a potential treatment of DED and appeared to be effective on both signs and symptoms of DED [15,16,17]. Indeed, a higher dietary intake of n-3 PUFAs was associated with the decrease of DED symptoms, while an unbalanced n-6 to n-3 PUFAs ratio was associated with a higher risk of DED [18]. However, as DED is a multifactorial disease, the potential role of plasma fatty acids (FAs) should be considered among other risk factors, especially in the elderly population [19,20].

Therefore, we aimed to investigate the association of plasma FAs and DED in a population-based study of participants aged 75 years and over and then to identify a FAs pattern more prone to be associated with DED.

## 2. Methods

### 2.1. Population Study

The Montrachet study (Maculopathy Optic Nerve and nuTRition neurovAsCular and HEarT) is an ancillary study of the population-based Three Cities (3C) study, which has previously been described [21]. Briefly, the 3C cohort study was undertaken to assess the relationship between vascular risk factors and aging disorders. Overall, 9294 persons aged 65 years and over, selected from the electoral rolls of three French urban cities (Bordeaux, Dijon and Montpellier), were included (*n* = 4931 living in Dijon). Ten years later, the subgroup of participants from Dijon were asked to participate in the Montrachet study in order to assess associations between age-related eye disorders and neurologic and heart diseases in the elderly. The methodology of the Montrachet study and the baseline characteristics of participants have already been thoroughly described [22].

A comprehensive ocular surface assessment (namely fluorescein tear film breakup time (TFBUT), Schirmer I test and fluorescein corneal staining) was performed [23], and plasma FAs were measured. Subjects with one parameter missing were excluded. Our main variable of interest was the presence of DED. Secondary variables of interest corresponded to plasma FAs composition. Covariates, including demographic, lifestyle, clinical and treatment variables, were documented in another recent paper [6]. More specifically, oral medication use (anxiolytic drugs and global psychotropics) was defined from an anatomic therapeutic chemical classification for drug use. Written informed consent was obtained from all participants. The study followed the tenets of the Declaration of Helsinki and was approved by the regional ethics committee and registered as 2009-A00448-49.

### 2.2. DED Evaluation and Definition

The evaluation and definition of DED signs were previously described in details [6]. In the present study, we only used clinical signs to define DED subjects, because signs and symptoms of DED are poorly correlated [24,25]. Briefly, participants presenting two out of the three following signs were considered as having DED: positive corneal staining, TFBUT < 5 s or Schirmer I test < 5 mm

### 2.3. Plasma Lipids Measurement

Lipids were extracted from plasma samples from fasted volunteers according to Moilanen and Nikkari [26]. Lipid extracts were stored under inert gas until further analyses. Total lipids from plasma were transmethylated using boron trifluoride in methanol according to Morrison and Smith [27]. Fatty acid methyl esters (FAMEs) were subsequently extracted with hexane and analyzed on a Hewlett Packard Model 5890 gas chromatograph (Palo Alto, CA, USA) using a CPSIL-88 column (100 m × 0.25 mm i.d., film thickness 0.20 µm) (Varian, Les Ulis, France) equipped with a flame ionization detector. Hydrogen was used as carrier gas (inlet pressure 210 kPa). The oven temperature was held at 60 °C for 5 min, increased to 165 °C at 15 °C/min and held for 1 min, and then, it was increased to 225 °C at 2 °C/min and finally held at 225 °C for 17 min. The injector and the detector were maintained at 250 °C. FAMEs were identified by comparison with commercial and synthetic standards. The data were processed using the EZChrom Elite software (Agilent Technologies, Massy, France) and reported as a percentage of the total FAs. For analysis, 25 plasma FAs (saturated, monounsaturated and polyunsaturated) levels were considered. Long-chain FAs were synthesized through desaturation and elongation; then, enzymatic activities indexes were estimated through the calculation of the precursor to product ratios of individuals FAs as follows: Δ5 desaturase (C20:4 n-6 to C20:3 n-6) and ELOVL2/5 (C22:4 n-6 to C20:4 n-6 and C22:5 n-3 to C20:5 n-3).

### 2.4. Statistics

Continuous variables were expressed as the mean (SD) or median (interquartile range) according to their distribution and categorical variables as a number (%). Bivariate comparisons were performed with Student’s *t*-test or ANOVA test for continuous variables and Pearson’s Chi-squared or Fisher’s exact tests for categorical variables when appropriate. To assess a potential non-responders bias, we compared characteristics of participants and non-participants. In order to identify the FAs pattern associated with the presence of DED, the analysis was performed in two steps. First, we used the partial least squares regression generalized linear models method (PLS) [28]. Similar to a principal component analysis (PCA), PLS is a dimension reduction method that extracts a set of orthogonal factors called latent variables, which are used as predictors in the regression model [29,30]. PLS aims at reducing the dimension of predictor variables Xi (i.e., concentrations of 25 FAs) with the constraint of maximizing the covariance between Xi and response variable Y (i.e., the presence of DED). We retained the PLS component that was significantly associated with the presence of DED [31]. The identified FAs pattern was constructed further to a score by weighting each FA concentration with factor weight values. Individual factor scores were then categorized into quartiles. To interpret the FA pattern, we kept those FAs with absolute values of weights ≥ 0.20 [32]. In the first step of analysis, we performed a distribution of socio-demographics, lifestyle and medication use by quartiles of the FAs pattern scores. Second, a multivariable logistic regression analysis expressed as ORs and their 95% confidence interval was performed to determine whether the FAs pattern scores were significantly associated with the presence of DED. The lowest quartile of FAs pattern scores was defined as the reference group. Models were systematically adjusted for age and sex. A stepwise procedure was used to select a final model after adjustment for age, sex, educational level, iris color, best-corrected visual acuity (BCVA), age-related macular degeneration (AMD), systemic hypertension, anxiolytics, antihistamine eye drops, lipid-lowering drugs use, plasma HDL cholesterol and triglycerides.

Finally, in order to evaluate the contribution of our plasma FAs pattern approach, we performed an analysis between clinically relevant FAs considered individually and DED using multivariable logistic regression models. For all analyses, the tests were two-sided, and results were considered significant when *p* < 0.05. Analyses were performed using SAS software (version 9.4; SAS institute Inc.; Cary, NC, USA) and R version 3.4.2 (plsRglm package) (http://www.R-project.org/, accessed on 20 April 2022).

## 3. Results

### 3.1. Demographic, Lifestyle and Clinical Characteristics of Participants

Among the 1153 participants of the Montrachet study, 740 subjects (mean [SD] age 82.2 [3.7] years, 62.7% women) had available clinical data and plasma FAs data for analysis (Fig.). The participants and non-participants presented the same demographic, lifestyle and clinical characteristics (Table 1). DED according to our definition (clinical sign-based DED) was present in 35.0% [95% CI 31.6–38.4] of participants. Table 2 presents the characteristics of participants and the age- and sex-adjusted associations according to DED presence. Participants with a short secondary school level, with a dark iris, using antianxiolytic drugs and with artificial tears were more likely to present DED, whereas participants with AMD were less likely to present DED.

### 3.2. Plasma Fatty Acids and DED

Table 3 presents the levels of plasma FAs and DED at inclusion (percentage of the total fatty acids). The highest levels of FAs found in the study participants were linoleic acid C18:2 n-6 (25.2%), palmitic acid C16:0 (22.3%), oleic acid C18:1 n-9 (21.4%) and arachidonic acid C20:4n-6 (AA, 7.7%). In the bivariate analysis, no difference in plasma FAs concentrations was found between subjects with and without DED. We identified a plasma FAs pattern associated with the presence of DED. This pattern explained 10.7% of the total variance in the original set of FAs and displayed high negative weights of gamma-linoleic acid (GLA, C18:3 n-6), arachidonic acid (AA, C20:4 n-6) and eicosapentaenoic acid (EPA, C20:5 n-3) PUFAs and stearic acid SFAs (C18:0). These FAs concentrations decreased significantly across quartiles of pattern scores except GLA and AA. On the contrary, this pattern presented high positive weights of DGLA (C20:3 n-6), DTA (C22:4 n-6), DPA n-6 (C22:5 n-6) PUFAs, vaccenic acid (C18:1 n-7) and oleic acid (C18:1 n-9) MUFAs and behenic acid (C22:0) SFAs. The concentrations of these FAs increased significantly across the quartiles of pattern scores (Table 4 and Supplementary Materials Table S1).

### 3.3. Demographic, Lifestyle and Medical Characteristics, DED and FAs Pattern Scores

The distribution of potential confounders by quartiles of the FAs pattern scores is shown in Table S2. Individuals in upper quartiles of pattern scores were more likely men, overweight, smokers, with AMD diagnosis, with more systemic drugs (diuretics, beta-blockers and lipid-lowering drug), a with higher plasma level of triglycerides and with lower plasma level of HDL cholesterol compared to those in lower quartiles.

Table 5 presents the associations of FAs pattern scores by quartiles and the presence of DED. We observed a positive and significant association between FAs pattern scores and DED (OR_crude_ 2.53 [95% CI 1.60–3.99], *p*-trend < 0.001). These findings remained statistically significant after controlling for age and sex (OR 2.45 [95% CI 1.55–3.88], *p*-trend < 0.001). Similar results were found after further adjustment for potential confounders (*p*-trend = 0.001). Indeed, individuals in the upper quartiles compared with those in the lower quartiles of FAs pattern scores were more likely to present DED (OR_adj_ 2.46 [95% CI 1.51–4.01]). Finally, when FAs were considered individually, associations appeared weaker than the relation between the pattern score and DED (Table S3), suggesting that our pattern approach with the FAs combination captured more information for the prediction of DED.

## 4. Discussion

While FAs plasma levels taken individually were not different in DED patients compared to non-DED subjects, we identified a plasma FAs pattern positively and significantly associated with DED: low concentrations of PUFAs (GLA, AA and EPA), high MUFAs (vaccenic and oleic acids) and low SFAs (i.e., stearic acid). Individuals in the upper quartiles of the FAs pattern scores were more likely to present DED than those in the lower quartiles. We found that the FAs combination depicted by this pattern was significantly associated with DED in our old population.

In a recent observational longitudinal study, the nutrients pattern characterized by low plasma PUFAs (LA, AA and EPA), low vitamin D and carotenes was significantly associated with a higher risk of dementia in elderly patients [33]. Observational studies have reported that n-3 PUFAs intake is implicated in the prevention of age-related macular degeneration [34,35]. A good balance between higher n-3 PUFAs and lower linoleic acid intakes could improve the incorporation rate in the neurosensory retina [36]. Moreover, a potential interaction between the complement system and lipoprotein homeostasis has been hypothesized [37]. In DED, the rationale for dietary recommendations with n-3 PUFAs is based on different features: (1) n-3 and n-6 PUFAs play an important role in inflammatory pathways [38,39]; (2) there is a competition between n-3 and n-6 long chain PUFAs metabolism with n-3 acting as competitor to decrease AA production [36]; (3) an increased production of inflammatory mediators like cytokines has been demonstrated in DED [40]. The American Academy of Ophthalmology and the guidelines from the DEWS still stated that nutritional supplementation with n-3 and n-6 PUFAs can be recommended in DED [17]. However these practices remain controversial: while a meta-analysis reported a discrete improvement in the parameters of tear function, a recent multicenter, double-blind clinical trial did not find better outcomes with n-3 fatty acid supplementation [41]. Indeed, as observed in age-related macular degeneration, lipid supplementation failed to demonstrate clinical efficacy in DED [42]. However, rather than the effect of one specific member of the PUFAs family, the anti-inflammatory effect in DED is probably the result of a subtle balance between three FAs that are precursors of bioactive molecules belonging to eicosanoids: dGLA, AA and EPA [18,43,44].

**Figure 1:**
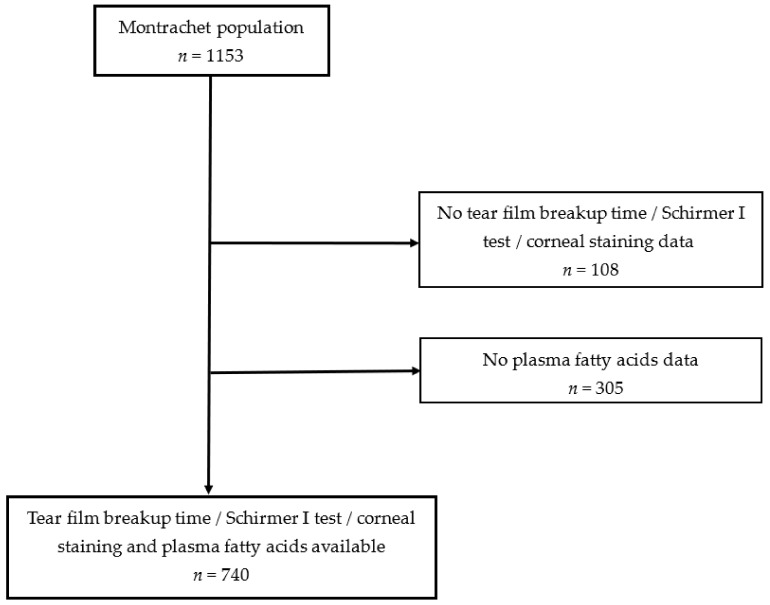
Workflow of fatty acids and dry eye disease in the Montrachet study.

The FAs pattern in this study is characterized by low PUFAs, especially low plasma concentrations of GLA, AA and EPA, and high concentrations of dGLA. GLA, AA and EPA have been reported to have implications in the DED pathogenesis when considered individually [20,45,46]. Additionally, it has been shown that these PUFAs improved the clinical signs of DED in an experimental rat model [44]. LA and ALA are the primary metabolites of n-6 and n-3 PUFAs, respectively (Figure 1). They are further elongated into dGLA and EPA, the last ones being precursors of anti-inflammatory eicosanoids from series-1 and series-2, respectively [47]. A lower plasma levels of GLA found in DED subjects can result either from the reduction of LA metabolization into GLA by Δ6-desaturase or from an increased conversion of GLA into dGLA by ELOVL5. Whereas the involvement of Δ6-desaturase and ELOVL5 activities remain unclear regarding the levels of GLA, the increased concentrations of dGLA are probably subsequent to the known inhibitory action of EPA on Δ5-desaturase. It can result in the accumulation of dGLA in the detriment of AA. Such a pattern may have consequences on the synthesis of dGLA-, AA- and EPA-derived eicosanoids, by favoring the synthesis of anti-inflammatory series-1 eicosanoids (derived from dGLA). On the contrary, it limits anti-inflammatory synthesis series-3 and pro-inflammatory series-2 eicosanoids (derived from EPA and AA, respectively). As inflammation plays a key role in DED pathophysiology [40], one can hypothesize that the increased conversion of dGLA into anti-inflammatory eicosanoids is not sufficient to balance the effects of the AA-derived pro-inflammatory eicosanoids. However, further investigations are needed.

Apart from the main PUFAs associated with DED above, our FAs pattern was also characterized by low plasma stearic acid, which has not been reported before, followed by high oleic acid and high vaccenic acid levels. These FAs are mainly dependent on a rich saturated and monounsaturated intake. The effect of SFAs on inflammation remain controversial [48,49]. The inverse association between stearic acid and inflammation had already been reported in young adults in whom circulating stearic acid was inversely associated with high-sensitivity C-reactive protein [50]. Indeed, a recent study conducted with in vitro and in vivo models of joint inflammation, showed anti-inflammatory effects of copolymers of palmitic, oleic and stearic acids on cartilage disease [51].

The statistically significant association between DED and AMD presented in Table 2 (*p* = 0.037) was not reported in other population-based studies. Besides the age-related nature of both conditions, AMD and DED, we do not think this represents a relevant clinical association.

We acknowledge several limitations in this study. First, the cross-sectional design of the study did not allow us to determine longitudinal associations between alterations of plasma FAs levels and DED. Second, the corneal staining was not measured quantitatively as in the Oxford classification. Third, some biological markers assessment could have been of special interest, for example, the PGE1 essay or enzyme expression analysis through an mRNA assay, but they were not explored in our study. Fourth, these results were found in our urban and healthy elderly population and cannot be generalized to other populations.

The strengths of this study include its large population sample size on the population-based design. Second, major confounding factors were taken into account in the multivariable analysis. Third, we investigated the association of a combination of plasma FAs with DED in this elderly population, while the previous studies were often based on specific FAs.

## 5. Conclusions

In conclusion, our work highlights that a plasma FAs pattern characterized by low PUFAs (GLA, AA, EPA), high MUFAs (vaccenic and oleic acids) and low saturated FAs (i.e., stearic acid) was significantly associated with DED in our elderly population after taking into account major known confounding factors. We showed that the PUFAs metabolism, as expressed by their estimated enzymatic activities indexes, has a clinical implication in DED.

## Figures and Tables

**Table 1:** Comparison of participants and non-participants for the association between plasma fatty acids and dry eye disease in the Montrachet Study, *n* = 1153.

	Non-Participants (*n* = 413)	Participants (*n* = 740)	
			*p*-Value
Age, years			
<80	144 (34.9)	256 (34.6)	0.134
80–85	161 (39.0)	325 (43.9)	
>85	108 (26.1)	159 (21.5)	
Sex, female	259 (62.7)	464 (62.7)	0.997
Body Mass Index, ≥25 kg/m^2^	210 (50.8)	345 (46.6)	0.168
Smoking status, former and current smokers	129 (32.1)	261 (35.7)	0.220
Alcohol consumption, yes	21 (6.0)	43 (6.5)	0.755
Education level			
No education or primary school	112 (27.2)	212 (28.6)	0.346
Short secondary school	63 (15.3)	97 (13.1)	
Long secondary school	65 (15.8)	142 (19.3)	
Post-secondary or university	172 (41.7)	289 (39.0)	
Sun protection			
Never	32 (7.8)	83 (11.2)	0.075
Occasionally	103 (25.2)	155 (20.9)	
Often	274 (67.0)	501 (67.9)	
Best-corrected visual acuity, <20/60	12 (2.9)	18 (2.4)	0.628
Central corneal thickness, µm	555.7 (35.6)	554.0 (34.9)	0.446
Iris color			
Blue/Gray	161 (39.0)	304 (41.1)	0.413
Green/Brown	139 (33.7)	221 (29.9)	
Dark Brown	113 (27.3)	215 (29.0)	
Medical history ^a^			
Systemic hypertension	226 (54.7)	448 (60.5)	0.054
Diabetes	33 (9.4)	60 (9.0)	0.858
Ocular history ^a^			
Age-related macular degeneration	12 (3.2)	28 (4.0)	0.477
Diabetic retinopathy	2 (0.5)	7 (0.9)	0.393
Glaucoma	50 (12.1)	87 (11.8)	0.849
Ocular hypertension	16 (3.8)	22 (3.0)	0.411
Cataract extraction	206 (50.2)	361 (48.8)	0.635
Systemic drugs			
Antihypertensives	210 (59.7)	405 (60.9)	0.699
Diuretics	59 (14.3)	99 (13.4)	0.667
Beta-blockers	95 (23.0)	177 (23.9)	0.725
Calcium antagonists	59 (14.3)	103 (13.9)	0.864
Anxiolytics	50 (12.1)	88 (11.9)	0.914
Global psychotropics	103 (24.9)	184 (24.9)	0.978
Antihistamines	21 (5.6)	37 (5.0)	0.949
Decongestants and antiallergics agents	3 (0.7)	11 (1.5)	0.258
Lipid-lowering drugs	133 (37.8)	291 (43.7)	0.066
Topical treatment			
Artificial tears	55 (13.3)	91 (12.3)	0.617
IOP-lowering agents			
Preserved eye drops	41 (9.9)	62 (8.4)	0.567
Non-preserved eye drops	7 (1.7)	10 (1.3)	
Antihistamine eye drops	55 (13.3)	91 (12.3)	0.617
Other eye drops	4 (1.0)	7 (0.9)	0.969
Plasma lipids, mmol/L mean (SD)			
Total cholesterol	5.81 (0.9)	5.79 (0.9)	0.665
LDL cholesterol	3.60 (0.8)	3.59 (0.8)	0.910
HDL cholesterol	1.67 (0.4)	1.66 (0.4)	0.774
Triglycerides	1.19 (0.5)	1.16 (0.5)	0.779

IOP = Intraocular pressure; LDL = Low density lipoprotein; HDL = High density lipoprotein; Missing data for smoking (*n* = 20), alcohol (*n* = 136), educational level (*n* = 1), sun protection (*n* = 5), central corneal thickness (*n* = 7), diabetes (*n* = 137), age-related macular degeneration (*n* = 84), lipid-lowering drugs (*n* = 136), antihypertensives (*n* = 136) and plasma lipids (*n* = 14). ^a^ self-reported, Secondary school levels: short (to age 15 years); long (to age 18 years). Values are given as a number (percentage) for categorial variables.

**Table 2:** Characteristics of participants and age- and sex-adjusted associations according to the presence of dry eye disease, *n* = 740.

			Dry Eye Disease				
			No (*n* = 481)	Yes (*n* = 259)	*p*-Value	OR (95% CI) ^b^	*p*-Value
Age, y							
<80			175 (36.4)	81 (31.3)	0.304	1.00 (reference)	
80–85			202 (42.0)	123 (47.4)		1.32 (0.93–1.86) ^c^	0.117
>85			104 (21.6)	55 (21.3)		1.15 (0.76–1.75) ^c^	0.509
Sex, female vs. male			305 (63.4)	159 (61.3)	0.587	0.91 (0.67–1.25) ^d^	0.572
Body Mass Index (≥25 kg/m^2^)			220 (45.7)	125 (48.3)	0.511	1.12 (0.82-1.52)	0.470
Smoking status, former and current smokers			167 (35.2)	94 (36.7)	0.674	1.06 (0.74–1.51)	0.742
Alcohol consumption, yes			25 (5.2)	18 (6.9)	0.715	1.03 (0.53–2.00)	0.920
Education level					0.157		
No education or primary school			140 (29.1)	72 (27.9)		1.09 (0.75–1.60)	0.651
Short secondary school			54 (11.2)	43 (16.6)		1.75 (1.09–2.8)	0.020
Long secondary school			90 (18.7)	52 (20.0)		1.25 (0.81–1.91)	0.310
High school or university			197 (41.0)	92 (35.5)		1.00 (reference)	
Sun protection					0.584		
Never			56 (11. 6)	27 (10.4)		0.85 (0.52–1.39)	0.523
Occasionally			105 (21.9)	50 (19.3)		0.82 (0.58–1.25)	0.408
Often			319 (66.5)	182 (70.3)		1.00 (reference)	
Best-corrected visual acuity, <20/60			12 (2.5)	6 (2.3)	0.881	0.84 (0.31–2.29)	0.733
Central corneal thickness, µm			555.6 (32.7)	551.2 (38.6)	0.101	0.99 (0.99–1.00)	0.136
Iris color					<0.0001		
Green/Brown			145 (31.1)	76 (29.3)		1.44 (0.98–2.09)	0.059
Dark brown			114 (23.7)	101 (39.0)		2.45 (1.69–3.55)	<0.0001
Blue			222 (46.1)	82 (31.7)		1.00 (reference)	
Medical history ^a^							
Systemic hypertension			280 (58.2)	168 (64.9)	0.077	1.29 (0.94–1.79)	0.113
Diabetes			34 (7.8)	26 (11.4)	0.117	1.54 (0.89–2.64)	0.119
Ocular history ^a^							
Age-related macular degeneration			23 (5.1)	5 (2.1)	0.051	0.35 (0.13–0.94)	0.037
Diabetic retinopathy			4 (0.8)	3 (1.2)	0.661	1.41 (0.31–6.35)	0.654
Glaucoma			46 (9.6)	34 (13.1)	0.136	1.21 (0.71–1.91)	0.425
Ocular hypertension			9 (1.8)	13 (5.0)	0.016	1.43 (0.78–2.61)	0.242
Cataract extraction			229 (47.6)	132 (60.0)	0.384	1.10 (0.80–1.50)	0.559
Systemic drugs							
Antihypertensives			254 (58.1)	151 (66.2)	0.042	1.39 (0.99–1.94)	0.058
Diuretics			60 (12.5)	39 (15.1)	0.325	1.21 (1.78–1.88)	0.395
Beta-blockers			108 (22.4)	69 (26.6)	0.203	1.22 (0.86–1.73)	0.265
Calcium antagonists			61 (12.7)	42 (16.2)	0.185	1.29 (0.84–1.98)	0.241
Anxiolytics			49 (10.2)	39 (15.1)	0.051	1.58 (1.00–2.49)	0.049
Global psychotropic			114 (23.7)	70 (27.0)	0.318	1.22 (0.86–1.73)	0.275
Antihistamines			23 (4.8)	14 (4.4)	0.710	1.15 (0.58–2.28)	0.687
Decongestants and anti-allergic agents			7 (1.5)	4 (1.5)	0.710	0.97 (0.28–3.37)	0.962
Lipid-lowering drugs			191 (43.7)	100 (43.9)	0.970	1.00 (0.73–1.39)	0.976
Topical treatment							
Artificial tears			50 (10.4)	41 (15.8)	0.032	1.64 (1.05–2.56)	0.029
IOP-lowering agents							
Preserved eye drops			41 (8.5)	21 (8.1)	0.598	0.94 (0.54–1.62)	0.813
Nonpreserved eye drops			5 (1.0)	5 (1.9)		1.89 (0.54–6.61)	0.320
Antihistamines eye drops			27 (5.6)	15 (5.8)	0.920	1.01 (0.53–1.95)	0.969
Other eye drops			6 (1.2)	1 (0.4)	0.248	0.33 (0.04–2.75)	0.305
Plasma lipid, mmol/L, mean (SD)							
Total cholesterol			5.8 (0.9)	5.8 (0.9)	0.888	1.01 (0.85–1.19)	0.932
LDL cholesterol			3.6 (0.8)	3.6 (0.8)	0.650	0.97 (0.81–1.17)	0.750
HDL cholesterol			1.7 (0.4)	1.6 (0.4)	0.747	0.97 (0.64–1.48)	0.905
Triglycerides			1.1 (0.5)	1.2 (0.5)	0.114	0.97 (0.95–1.72)	0.108

OR = Odds ratio; CI = Confidence interval; IOP = Intraocular pressure; LDL = Low density lipoprotein; HDL = High density lipoprotein, Secondary school levels: short (to age 15 years); long (to age 18 years). Missing data for smoking (*n* = 4), alcohol (*n* = 75), sun protection (*n* = 1), central corneal thickness (*n* = 2), diabetes (*n* = 76), age-related macular degeneration (*n* = 48), Lipid lowering drug use (*n* = 75), antihypertensives drug use (*n* = 75) and plasma lipids (*n* = 9). Values are given as a number (percentage) for categorial variables. ^a^ Self-reported. ^b^ Adjustment for age and sex. ^c^ Sex-adjusted OR. ^d^ Age-adjusted OR. Statistically significant *p*-values are in bold.

**Table 3:** Percentages of plasma fatty acids according to the presence of dry eye disease, *n* = 740.

			Dry Eye Disease		
Chemical Structure	Common Name	All FAs	No *n* = 481	Yes *n* = 259	*p*-Value
SFAs					
C12:0	Lauric acid	0.4	0.5	0.5	0.476
C14:0	Myristic acid	1.5	1.2	1.4	0.640
C15:0	Pentadecanoic acid	0.4	0.5	0.5	0.965
C16:0	Palmitic acid	22.3	22.3	22.2	0.602
C17:0	Margaric acid	0.5	0.3	0.3	0.654
C18:0	Stearic acid	7.2	7.0	6.8	0.156
C20:0	Arachidic acid	0.6	0.4	0.4	0.645
C22:0	Behenic acid	0.2	0.5	0.4	0.134
MUFAs					
C16:1 n-7	Palmitoleic acid	2.7	2.8	2.8	0.976
C16:1 n-9	Cis-7 hexadecenoic acid	0.6	0.8	0.6	0.578
C18:1 n-7	Vaccenic acid	2.1	2.1	2.2	0.396
C18:1 n-9	Oleic acid	21.4	21.5	21.5	0.341
C20:1 n-9	Eicosenoic acid	0.2	0.3	0.3	0.372
C24:1 n-9	Nervonic acid	0.4	0.4	0.5	0.644
PUFAs					
Omega-6					
C18:2 n-6	Linoleic acid (LA)	25.2	25.0	25.2	0.212
C18:3 n-6	Gamma linoleic acid (GLA)	0.6	0.5	0.6	0.831
C20:2 n-6	Eicosadienoic acid	0.3	0.5	0.3	0.052
C20:4 n-6	Arachidonic acid (AA)	7.7	7.4	7.5	0.265
C22:4 n-6	Docosatetraenoic Acid (DTA)	0.3	0.4	0.3	0.089
C22:5 n-6	Docosapentaenoic n-6 (DPA)	0.1	0.3	0.2	0.642
Omega-3					
C18:3 n-3	Alpha linoleic acid (ALA)	0.8	0.8	0.7	0.162
C20:5 n-3	Eicosapentaenoic acid (EPA)	1.4	1.3	1.4	0.271
C22:5 n-3	Docosapentaenoic n-3 (DPA)	0.7	0.7	0.8	0.630
C22:6 n-3	Docosahexaenoic acid (DHA)	2.4	2.5	2.6	0.476

FAs = Fatty acids; SFAs = Saturated Fatty acids; MUFAs = Monounsaturated Fatty acids; PUFAs = Polyunsaturated Fatty acids; GLA = Gamma-linoleic acid; DPA = Docosapentaenoic acid; EPA = Eicosapentaenoic acid; DHA = Docosahexaenoic acid. The results are reported as a percentage of the total fatty acids.

**Table 4:** Factor weight values of plasma fatty acids obtained by the partial least squares regression generalized linear models associated with the presence of dry eye disease in the Montrachet Study.

Fatty Acids	Common Name	Component ^a^
SFAs		
C12:0	Lauric acid	0.13
C14:0	Myristic acid	0.07
C15:0	Pentadecanoic acid	0.01
C16:0	Palmitic acid	0.11
C17:0	Margaric acid	0.09
C18:0	Stearic acid	−0.32
C20:0	Arachidic acid	0.09
C22:0	Behenic acid	0.27
MUFAs		
C16:1 n-7	Palmitoleic acid	0.09
C16:1 n-9	Cis-7 hexadecenoic acid	−0.00
C18:1 n-7	Vaccenic acid	0.39
C18:1 n-9	Oleic acid	0.20
C20:1 n-9	Eicosenoic acid	0.17
C24:1 n-9	Nervonic acid	0.19
PUFAs		
Omega-6		
C18:2 n-6	Linoleic acid (LA)	−0.10
C18:3 n-6	Gamma linoleic acid (GLA)	−0.27
C20:2 n-6	Eicosadienoic acid	0.05
C20:3 n-6	Dihomo-gamma-linoleic acid (DGLA)	0.29
C20:4 n-6	Arachidonic acid (AA)	−0.21
C22:4 n-6	Docosatetraenoic acid (DTA)	0.22
C22:5 n-6	Docosapentaenoic acid n-6 (DPA)	0.32
Omega-3		
C18:3 n-3	Alpha linoleic acid (ALA)	−0.08
C20:5 n-3	Eicosapentaenoic acid (EPA)	−0.27
C22:5 n-3	Docosapentaenoic acid n-3 (DPA)	0.21
C22:6 n-3	Docosahexaenoic acid (DHA)	−0.07

SFAs = Saturated Fatty acids; MUFAs = Monounsaturated Fatty acids; PUFAs = Polyunsaturated Fatty acids. ^a^ The FAs pattern obtained from plsRglm explained 10.74% of the variance in all FAs, and values in bold indicate interpretable FAs with absolute values of weights ≥ 0.20.

**Table 5:** Multivariable associations of plasma fatty acids pattern scores across quartiles and the presence of dry eye disease in the Montrachet study.

	Quartiles of FAs Pattern Scores				
	Q1	Q2	Q3	Q4	
	OR (95% CI)	OR (95% CI)	OR (95% CI)	OR (95% CI)	*p*-Trend
Crude	1.00 (Ref.)	2.31 (1.46–3.65)	2.26 (1.43–3.57)	2.53 (1.60–3.99)	<0.001
M1 ^a^	1.00 (Ref.)	2.30 (1.45–3.64)	2.22 (1.40–3.52)	2.45 (1.55–3.88)	<0.001
M2 ^b^	1.00 (Ref.)	2.44 (1.50–3.95)	2.26 (1.39–3.65)	2.46 (1.51–4.01)	0.001

Ref = Reference; OR = Odds ratio; CI = Confidence interval, Q1 = First quartile, Q2 = Second quartile, Q3 = Third quartile, Q4 = Fourth quartile. ^a^ M1: age- and sex-adjusted model. ^b^ M2: M1 with further adjustment for educational level, iris color, best-corrected visual acuity, age-related macular degeneration, systemic hypertension, anxiolytic, antihistamine eye drops, lipid-lowering drugs and plasma HDL cholesterol and triglycerides. Statistically significant *p*-values are in bold.

## Data Availability

Data are available on reasonable request. A 3C study data agreement request should be sent to louis.arnould@chu-dijon.fr.

## Supplementary Materials

The following are available online at https://www.mdpi.com/article/10.3390/nu14112290/s1: Table S1. Mean percentages of fatty acids by quartiles of fatty acids pattern scores (*n* = 740). Table S2. Demographics and lifestyle characteristics of study participants and fatty acids pattern scores distribution (*n* = 740). Table S3. Associations of plasma fatty acids and Dry Eye Disease in the Montrachet Study.
