# Supplementary material for: Plasma Fatty Acids Pattern and Dry Eye Disease in the Elderly: The Montrachet Population-Based Study
Source: Nutrients. 2022 May 30;14(11):2290. doi: 10.3390/nu14112290 (PMC9183164; doi:10.3390/nu14112290)
Supplement: Supplementary file 1 [file nutrients-14-02290-s001.zip › nutrients-1725879-supplementary.pdf]

Table S1.

Mean percentages of fatty acids by quartiles of fatty acids pattern scores (n = 740).

Chemical structure	Common name	Quartiles of FAs pattern scores				
		Q1	Q2	Q3	Q4	P-value
SFAs						
C12:0	Lauric acid	0.1 (0.1)	0.1 (0.1)	0.2 (0.1)	0.2 (0.1)	**<0.001**
C14:0	Myristic acid	1.1 (0.3)	1.1 (0.3)	1.2 (0.4)	1.2 (0.4)	**<0.001**
C15:0	Pentadecanoic acid	0.3 (0.1)	0.3 (0.1)	0.3 (0.1)	0.3 (0.1)	**<0.001**
C16:0	Palmitic acid	21.7 (1.5)	22.0 (1.6)	22.3 (1.9)	22.4 (1.8)	**<0.001**
C17:0	Margaric acid	0.3 (0.1)	0.3 (0.1)	0.3 (0.1)	0. 4 (0.1)	**<0.001**
C18:0	Stearic acid	7.2 (0.6)	7.0 (0.5)	6.8 (0.5)	6.6 (0.6)	**<0.001**
C20:0	Arachidic acid	0.1 (0.1)	0.1 (0.1)	0.1 (0.1)	0.2 (0.1)	**<0.001**
C22:0	Behenic acid	0.2 (0.1)	0.2 (0.1)	0.2 (0.1)	0.3 (0.1)	**<0.001**
MUFAs						
C16:1 n-7	Palmitoleic acid	2.2 (0.6)	2.5 (0.7)	2.8 (0.7)	3.2 (1.0)	**<0.001**
C16:1 n-9	Cis-7 hexadecenoic acid	0.4 (0.1)	0.4 (0.1)	0.4 (0.1)	0.4 (0.1)	**<0.001**
C18:1 n-7	Vaccenic acid	1.6 (0.2)	1.8 (0.2)	1.8 (0.2)	2.1 (0.3)	**<0.001**
C18:1 n-9	Oleic acid	19.9 (2.1)	21.2 (2.1)	21.8 (2.1)	22.5 (2.9)	**<0.001**
C20:1 n-9	Eicosenoic acid	0.2 (0.1)	0.2 (0.1)	0.2 (0.1)	0.3 (0.1)	**<0.001**
C24:1 n-9	Nervonic acid	0.3 (0.1)	0.4 (0.1)	0.4 (0.2)	0.5 (0.2)	**<0.001**
PUFAs						
Omega-6						
C18:2 n-6	Linoleic acid	27.4 (3.4)	25.5 (3.1)	24.4 (3.2)	22.6 (3.8)	**<0.001**
C18:3 n-6	Gamma linoleic acid	0.5 (0.2)	0.5 (0.2)	0.5 (0.2)	0.4 (0.1)	0.431
C20:2 n-6	Eicosadienoic acid	0.2 (0.1)	0.2 (0.1	0.2 (0.1)	0.3 (0.3)	**0.002**
C20:3 n-6	Dihomo-gamma-linoleic acid	1.4 (0.3)	1.5 (0.3)	1.6 (0.3)	1.6 (0.3)	**<0.001**
C20:4 n-6	Arachidonic acid	7.6 (1.4)	7.5 (1.4)	7.4 (1.4)	7.3 (1.6)	0.241
C22:4 n-6	Docosatetraenoic Acid	0.2 (0.1)	0.2 (0.1)	0.3 (0.1)	0.3 (0.1)	**<0.001**
C22:5 n-6	Docosapentaenoic n-6	0.1 (0.1)	0.2 (0.1)	0.2 (0.1)	0.2 (0.1)	**<0.001**
Omega-3						
C18:3 n-3	Alpha linoleic acid	0.6 (0.2)	0.6 (0.2)	0.6 (0.2)	0.6 (0.2)	0.412
C20:5 n-3	Eicosapentaenoic acid	1.5 (0.7)	1.3 (0.6)	1.2 (0.5)	1.1 (0.5)	**<0.001**
C22:5 n-3	Docosapentaenoic n-3	0.6 (0.1)	0.6 (0.1)	0.6 (0.1)	0.6 (0.1)	**<0.001**
C22:6 n-3	Docosahexaenoic acid	2.4 (0.6)	2.2 (0.6)	2.2 (0.6)	2.2 (0.6)	**<0.001**

SFAs = Saturated fatty acids; MUFAs = Monounsaturated fatty acids; PUFAs = Polyunsaturated fatty acids Q1: first quartile, Q2; second quartile, Q3: third quartile, Q4: fourth quartile. The results are expressed as mean (SD = standard deviation) of total fatty acids.

Statistically significant *P*-values are in bold.

Table S2.

Demographics and lifestyle characteristics of study participants and fatty acids pattern scores distribution (n = 740).

	Quartiles of fatty acids pattern scores				
	Q1	Q2	Q3	Q4	*P*-trend
Age, y	81.8 (3.7)	81.9 (3.5)	82.2 (3.5)	82.7 (3.9)	0.067
Sex, female vs male	127 (68.6)	119 (64.3)	108 (58.4)	110 (59.5)	**0.038**
Body Mass Index (> 25 kg/m²)	78 (42.2)	77 (41.6)	86 (46.5)	104 (56.2)	**0.004**
Smoking status, former and current smokers	61 (33.5)	52 (28.3)	73 (40.6)	75 (40.5)	**0.035**
Alcohol consumption, yes	9 (5.3)	9 (5.2)	15 (9.3)	10 (6.2)	0.438
Education level					0.083
No education or primary school	43 (23.3)	60 (32.4)	55 (29.7)	54 (29.2)	
Short secondary school	23 (12.4)	15 (8.1)	26 (14.1)	33 (17.8)	
Long secondary school	40 (21.6)	39 (21.1)	33 (17.8)	30 (16.3)	
High school or university	79 (42.7)	71 (38.4)	71 (38.4)	68 (36.7)	
Sun protection					0.172
Never	14 (7.6)	25 (13.5)	16 (8.6)	28 (15.1)	
Occasionnally	36 (19.6)	34 (18.4)	52 (28.2)	33 (17.8)	
Often	134 (72.8)	126 (68.1)	117 (63.2)	124 (67.1)	
Best-Corrected Visual Acuity, <20/60	2 (1.1)	6 (3.2)	4 (2.2)	6 (3.2)	0.339
Central corneal thickness, μm	555.7 (3.3)	554.2 (36.0)	550.2 (31.8)	556.2 (32.8)	0.352
Iris color					0.860
Green/Brown	73 (39.5)	75 (40.5)	79 (42.7)	77 (41.6)	
Dark brown	64 (34.6)	56 (30.3)	50 (27.0)	51 (27.6)	
Blue	48 (25.9)	54 (29.2)	56 (30.3)	57 (30.8)	
Medical history[a]					
Systemic hypertension	99 (53.5)	118 (63.8)	116 (62.7)	115 (62.2)	0.130
Diabetes	17 (10.1)	12 (6.9)	16 (9.9)	15 (9.4)	0.952
Ocular history[a]					
Age-related macular degeneration	2 (1.2)	9 (5.2)	6 (3.4)	11 (6.5)	0.037
Diabetic retinopathy	3 (1.6)	1 (0.5)	3 (1.6)		0.314
Glaucoma	18 (9.7)	25 (13.5)	19 (10.3)	25 (13.5)	0.475
Ocular hypertension	15 (8.1)	13 (7.0)	9 (4.9)	10 (5.4)	0.226
Cataract extraction	88 (47.6)	93 (50.3)	77 (41.6)	103 (55.7)	0.357
Systemic drugs					
Antihypertensives	91 (53.8)	108 (62.4)	163 (63.6)	103 (63.6)	0.064
Diuretics	19 (10.3)	20 (10.8)	27 (14.5)	33 (17.8)	**0.020**
Beta-blockers	31 (16.8)	44 (23.8)	50 (27.0)	52 (28.1)	**0.009**
Calcium antagonists	26 (14.0)	30 (16.2)	30 (16.2)	18 (9.7)	0.255
Anxiolytics	19 (10.3)	27 (14.6)	22 (11.9)	20 (10.9)	0.959

Global psychotropic	49 (26.5)	48 (25.9)	43 (23.2)	44 (23.8)	0.470
Antihistamines	7 (3.8)	12 (6.5)	4 (2.1)	14 (7.6)	0.367
Decongestants and anti-allergic agents	2 (1.1)	4 (2.2)	3 (1.6)	2 (1.1)	0.910
Topical treatment,					
IOP lowering agents					0.124
Preserved eye drops	12 (6.5)	17 (9.2)	12 (6.5)	21 (11.3)	
Unpreserved eye drops	1 (0.5)	4 (2.2)	1 (0.5)	4 (2.2)	
Antihistamine eye drops	9 (4.9)	13 (7.0)	9 (4.9)	11 (5.9)	0.943
Other eye drops	2 (1.1)	2 (1.1)	1 (0.5)	2 (1.1)	1.00
Lipid-lowering drugs	57 (33.7)	68 (39.3)	87 (53.7)	79 (49.1)	**<0.001**
Plasma lipid, µg/Ml					
Total cholesterol	5.9 (1.0)	5.8 (1.0)	5.7 (1.0)	5.7 (0.9)	0.067
LDL cholesterol	3.7 (0.8)	3.6 (0.9)	3.6 (0.8)	3.5 (0.8)	0.198
HDL cholesterol	1.8 (0.4)	1.7 (0.4)	1.6 (0.4)	1.6 (0.4)	**<0.0001**
Triglycerides	1.0 (0.4)	1.1 (0.5)	1.2 (0.5)	1.2 (0.6)	**<0.0001**

Values are given as number (percentage) for categorial variables.

IOP = Intraocular pressure; LDL = Low density lipoprotein; HDL = High density lipoprotein

Q1= First quartile; Q2 = Second quartile; Q3 = Third quartile; Q4 = Fourth quartile.

Missing data for smoking (n = 4), alcohol (n = 75), sun protection (n = 1), central corneal thickness (n = 2), diabetes (n=76), age-related macular degeneration (n = 48), lipid-lowering drugs (n = 75), Antihypertensives (n = 75) and plasma lipids (n = 9).

[a] Self-reported. Statistically significant *P*-values are in bold.

Table S3.

Associations of plasma fatty acids and dry eye disease in the Montrachet Study.

Chemical structure	Common name	Dry Eye Disease			
		OR (95%CI)[a]	P-trend[b]	OR (95%CI)[c]	*P*-trend[d]
SFAs					
C18:0	Stearic acid				
	Q1	1.00 (reference)	0.165	1.00 (reference)	0.307
	Q2	0.70 (0.45-1.07)		0.73 (0.46-1.16)	
	Q3	0.90 (0.59-1.36)		0.98 (0.62-1.50)	
	Q4	0.65 (0.42-1.02)		0.69 (0.43-1.11)	
PUFAs					
Omega-6					
C18:3 n-6	Gamma-linoleic Acid				
	Q1	1.00 (reference)	0.366	1.00 (reference	0.294
	Q2	0.95 (0.60-1.51)		0.99 (0.61-1.60)	
	Q3	0.91 (0.61-1.37)		0.88 (0.57-1.37)	
	Q4	0.82 (0.52-1.27)		0.79 (0.49-1.27)	
C20:3 n-6	Dihomo-gamma-linoleic acid				
	Q1	1.00 (reference)	0.003	1.00 (reference)	**0.002**
	Q2	0.84 (0.54-1.32)		0.96 (0.60-1.55)	
	Q3	1.45 (0.93-2.24)		1.62 (1.01-2.58)	
	Q4	1.70 (1.08-2.66)		1.87 (1.16-3.01)	
C20:4 n-6	Arachidonic acid				
	Q1	1.00 reference)	0.198	1.00 (reference)	0.282
	Q2	1.22 (0.80-1.87)		1.28 (0.81-2.01)	
	Q3	1.03 (0.66-1.55)		1.11 (0.70-1.74)	
	Q4	0.78 (0.51-1.22)		0.79 (0.49-1.28)	
C22:4 n-6	Docosatetraenoic acid				
	Q1	1.00 (reference)	0.181	1.00 (reference)	0.205
	Q2	1.48 (0.95-2.31)		1.40 (0.87-2.24)	
	Q3	1.29 (0.83-2.01)		1.22 (0.76-1.94)	
	Q4	1.48 (0.94-2.34)		1.48 (0.91-4.42)	

Chemical structure	Common name	OR (95%CI)[a]	P-trend[b]	OR (95%CI)[c]	P-trend[d]
C22:5 n-6	Docosapentaenoic acid (DPA)				
	Q1	1.00 (reference)	0.045	1.00 (reference)	0.053
	Q2	1.26 (0.78-2.02)		1.47 (0.87-2.42)	
	Q3	1.59 (1.03-2.46)		1.68 (1.06-2.66)	
	Q4	1.52 (0.93-2.46)		1.60 (0.96-2.67)	
Omega-3					
C20:5 n-3	Eicosapentaenoic Acid (EPA)				
	Q1	1.00 (reference)	0.289	1.00 (reference)	0.339
	Q2	0.71 (0.46-1.10)		0.71 (0.45-1.12)	
	Q3	0.82 (0.54-1.25)		0.84 (0.54-1.32)	
	Q4	0.75 (0.49-1.15)		0.74 (0.47-1.18)	
C22:5 n-3	Docosapentaenoic Acid (DPA)				
	Q1	1.00 (reference)	0.209	1.00 (reference)	0.185
	Q2	0.85 (0.54-1.37)		0.83 (0.52-1.33)	
	Q3	1.09 (0.71-1.68)		1.07 (0.68-1.68)	
	Q4	1.23 (0.79-1.92)		1.27 (0.80-2.04)	
Enzymatic activities indexes					
C20:4 n-6 to C20:3 n-6	Δ5 desaturase FADS1				
	Q1	1.00 (reference)	0.048	1.00 (reference)	**0.033**
	Q2	0.69 (0.45-1.05)		0.67 (0.43-1.05)	
	Q3	0.68 (0.44-1.04)		0.64 (0.41-0.99)	
	Q4	0.64 (0.42-0.98)		0.61 (0.38-0.96)	
C22:4 n-6 to C20:4 n-6	ELOVL2/5				
	Q1	1.00 (reference)	0.013	1.00 (reference)	**0.009**
	Q2	1.12 (0.72-1.73)		1.15 (0.72-1.83)	
	Q3	0.91 (0.58-1.42)		0.95 (0.59-1.52)	
	Q4	1.83 (1.20-2.80)		2.02 (1.27-3.20)	
C22:5 n-3 to C20:5 n-3	ELOVL2/5				
	Q1	1.00 (reference)	0.045	1.00 (reference)	0.051
	Q2	0.93 (0.59-1.45)		0.92 (0.57-1.47)	

	Q3	1.27 (0.82-1.96)	1.33 (0.83-2.11)
	Q4	1.43 (0.93-2.20)	1.43 (0.90-2.27)

SFAs = Saturated fatty acids; PUFAs = Polyunsaturated fatty acids; DGLA = Dihomo-gamma-linoleic acid; DTA = Docosatetraenoic acid; DPA = Docosapentaenoic acid; ELOVL2/5 = Elongation of very long chain fatty acids protein 2/5; DED = Dry Eye Disease; OR = Odds ratio; Q1 = First quartile; Q2 = Second quartile; Q3 = Third quartile; Q4 = Fourth quartile.

[a] Age and sex adjusted ORs.

[b] Adjusted ORs for age, sex, educational level, iris color, best-corrected visual acuity, age-related macular degeneration, systemic hypertension, antianxiolytics, antihistamine drop; lipid lowering drug use, Plasma HDL cholesterol and triglycerides.

[c] P-trend for logistic regression calculated by using median values within each category of FAs after adjustment for age and sex.

[d] P-trend for logistic regression calculated by using median values within each category of FAs after adjustment for age, sex, educational level, iris color, best-corrected visual acuity, age-related macular degeneration, systemic hypertension, antianxiolytics, antihistamine eye drops; lipid-lowering drugs, Plasma HDL cholesterol and triglycerides. Statistically significant *P*-values are in bold.
